# High‐Resolution Single‐Neuron Reconstruction Analysis in Golgi‐Stained Brain Tissues

**DOI:** 10.1111/cpr.70092

**Published:** 2025-07-06

**Authors:** Qiaowei Tang, Binfu Fan, Xiaoqing Cai, Zhiming Shen, Jichao Zhang, Jun Hu, Jiang Li, Ying Zhu

**Affiliations:** ^1^ Institute of Materiobiology College of Sciences, Shanghai University Shanghai China; ^2^ Xiangfu Laboratory Jiashan China; ^3^ School of Physical Science and Technology ShanghaiTech University Shanghai China; ^4^ Shanghai Synchrotron Radiation Facility Shanghai Advanced Research Institute, Chinese Academy of Sciences Shanghai China; ^5^ Institute of Neuroscience, Key Laboratory of Brain Cognition and Brain‐Inspired Intelligence Technology, Center for Excellence in Brain Science and Intelligence Technology Chinese Academy of Sciences Shanghai China

**Keywords:** 3D reconstruction, fluorescence micro‐optical sectioning tomography (fMOST), Golgi, high‐fidelity, single neuron, synchrotron‐based X‐ray microscopy

## Abstract

Understanding the structural and functional organisation of brain networks is a fundamental objective in neuroscience, with three‐dimensional (3D) reconstruction of single‐neuron morphology serving as a critical foundation. The Golgi staining method, which enables random neuronal labeling and provides high‐contrast signals in both optical and X‐ray microscopy, remains a valuable tool for morphological analysis. However, its widespread application in large‐scale neuronal reconstructions is hindered by signal discontinuities in neuronal branches, high‐density labeling, and complex background interference. While automated reconstruction methods perform well in sparsely labelled and morphologically simple neuronal populations, their effectiveness is limited in Golgi‐stained samples. Here we develop a semi‐automated single‐neuron reconstruction method for Golgi‐stained mouse brain neurons (SNR‐Golgi). By integrating three key technical modules—background denoising, single‐neuron extraction, and branch repair—SNR‐Golgi significantly enhances the accuracy and completeness of neuronal reconstruction. In fluorescence micro‐optical sectioning tomography (fMOST) datasets, SNR‐Golgi demonstrated superior performance in neuronal reconstruction within the mouse somatosensory cortex, achieving a 30% increase in reconstructed branch count, a 76% improvement in total branch length, and a 3.7‐fold increase in axonal length. Additionally, in synchrotron‐based X‐ray imaging datasets, SNR‐Golgi enabled submicron‐resolution 3D reconstruction of single neurons. These results demonstrate that SNR‐Golgi effectively addresses the complexity of Golgi‐stained samples and provides robust technical support for the structural analysis of brain neurons across various imaging modalities.

## Graphical abstract

SNR‐Golgi, a semi‐automated single−neuron three‐dimensional reconstruction strategy for Golgi−stained samples, which integrates background denoising, single‐neuron extraction and branch repair, enables high‐fidelity single‐neuron tracing in brain tissues across both optical and X‐ray imaging modalities.

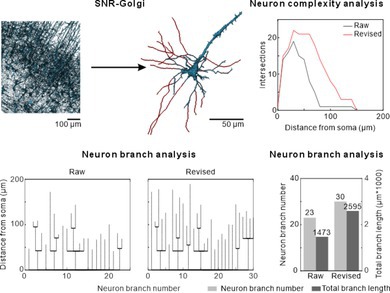

## 1 Introduction

Deciphering the structure and function of brain neuronal networks is a central objective in neuroscience, with neuronal morphology playing a crucial role in elucidating brain architecture and function [1, 2]. Golgi staining, which enables random labeling of neurons and provides high‐contrast visualisation of soma, dendrites, axons, and dendritic spines, has become a fundamental tool for morphological studies [3, 4]. For example, Chailangkarn et al. employed Golgi staining to analyse postmortem human cortical neurons in layers V/VI, revealing morphological alterations associated with Williams syndrome [5]. Similarly, Suzuki et al. used Golgi staining to demonstrate that hippocampal dendritic spine density, reduced in 5xFAD Alzheimer's disease model mice, could be restored upon CPTX (a synthetic synaptic organiser protein) injection, as observed via optical microscopy [6]. Furthermore, combining with synchrotron‐based X‐ray imaging, Hwu et al. applied Golgi staining for whole‐brain neuronal labeling in *Drosophila*, achieving 3D reconstruction of neuronal structures, demonstrating the compatibility of Golgi staining with high‐resolution X‐ray microscopy [7].

Despite these advantages, Golgi‐stained samples pose significant challenges for neuronal extraction and 3D reconstruction, particularly due to frequent signal discontinuities in neuronal branches [8, 9]. These discontinuities primarily arise from staining heterogeneity and incomplete dendritic labeling [10]. Additionally, while Golgi staining randomly labels ~1%–3% of neurons, the resulting density remains relatively high, complicating neuronal segmentation [11, 12]. Further challenges include incomplete tissue perfusion, mechanical artefacts from sectioning, and non‐specific staining of vasculature and glial cells, all of which exacerbate imaging noise and reconstruction errors [13]. These limitations hinder the comprehensive reconstruction of neuronal structures, potentially leading to the loss of critical network information and impeding functional analysis of complex brain circuits.

To address these challenges, various 3D neuronal reconstruction tools have been developed, broadly categorised into fully automated and semi‐automated methods [14, 15, 16]. Fully automated tools, such as NeuroGPS‐Tree and NeuronCyto II, rely on computational algorithms to reconstruct neuronal morphology with minimal user intervention, making them well‐suited for sparsely labelled and morphologically simple neurons. Gou et al. developed an automated system called Gapr accelerates projectome reconstruction (Gapr), which enables large‐scale neuron reconstruction from terabyte and even petabyte data [17]. However, in cases of weak signals or branch discontinuities, these methods often fail to ensure reconstruction integrity [18, 19, 20, 21]. Semi‐automated tools integrate user input with algorithmic computation to enhance reconstruction accuracy. While widely used tools such as Neuromantic, ManSegTool, and fast neurite tracer (FNT) perform well in ultra‐sparse fluorescently labelled samples, their efficacy diminishes in complex backgrounds with densely labelled Golgi‐stained neurons [15, 22, 23]. These tools struggle to differentiate neuronal structures from non‐neuronal elements and lack the capability to reconstruct neurons with significant signal interruptions. Thus, developing an approach that effectively restores interrupted signals and enables high‐fidelity single‐neuron reconstruction remains an urgent need in the field.

In this study, we develop single‐neuron reconstruction in Golgi‐stained brain tissues (SNR‐Golgi), a semi‐automated method tailored for neuronal reconstruction in Golgi‐stained samples. By applying SNR‐Golgi to multiple brain regions, including the visual cortex (VIS), somatosensory cortex (SS), and hippocampal cornu ammonis (CA) regions, we achieve accurate single‐neuron extraction and branch signal repair. The method demonstrates robust applicability and reliability across both fMOST and synchrotron‐based X‐ray imaging datasets.

## 2 Results and Discussion

### 2.1 Establishment of SNR‐Golgi Method

**FIGURE 1:**
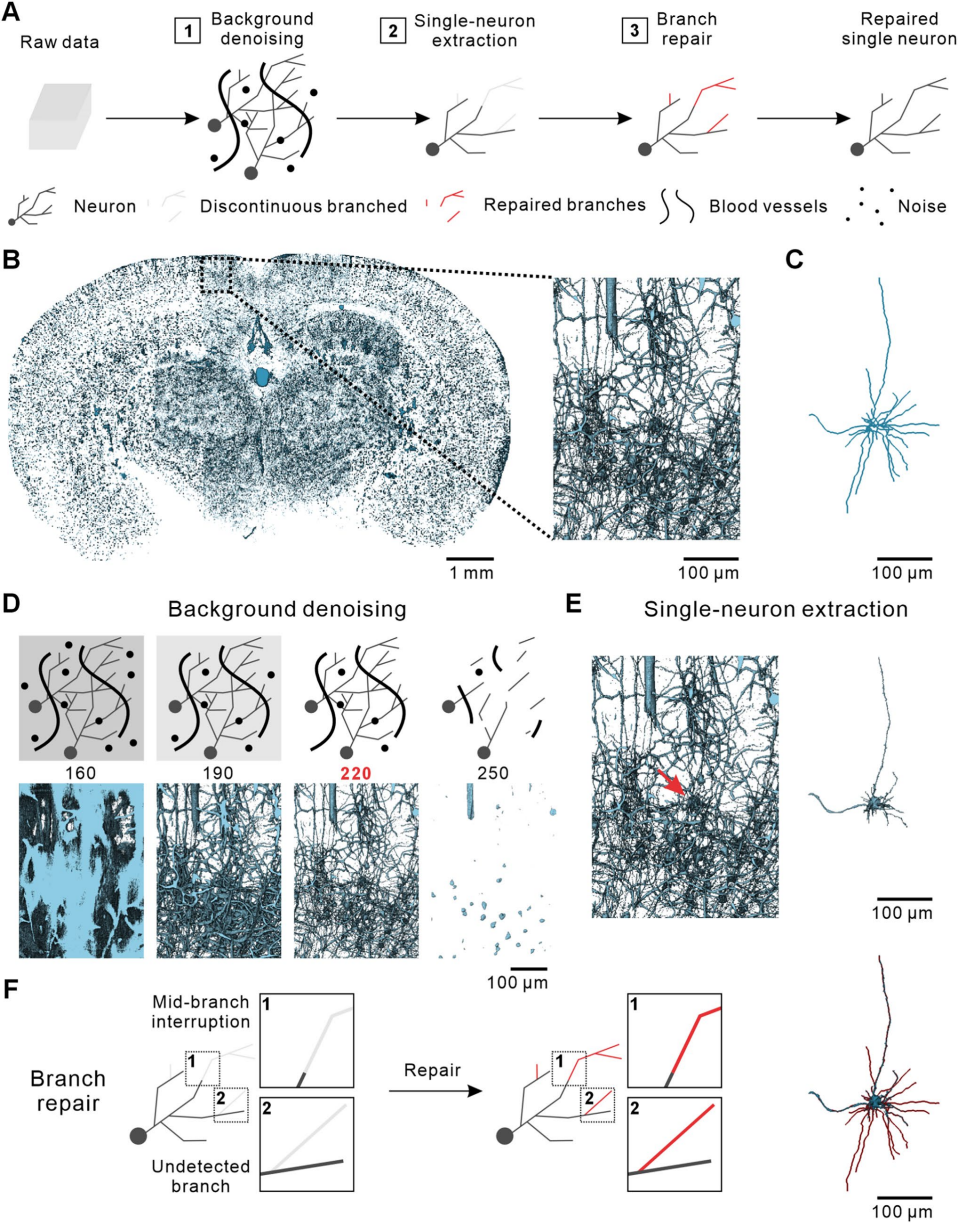
Establishment of SNR‐Golgi method. (A) Workflow of the SNR‐Golgi method. Raw data are processed through three modules to obtain the single neuron repaired. (B) Coronal section of the mouse brain and its magnified view. (C) Repaired single neuron extracted from (B). (D) Background denoising module. Top: Schematic illustration. Bottom: 3D visualisation of the denoised data. (E) Single‐neuron extraction module. Left: 3D visualisation of the data after background denoising. Right: Extraction of the neuron indicated by the red arrow in the left panel. (F) Branch repair module. Left: Schematic illustration of the repair for two types of branch discontinuities. Right: Repaired results of the single neuron shown in (E).

In designing the SNR‐Golgi method, we identified two major challenges in neuronal reconstruction from Golgi‐stained samples: (1) nonspecific staining of blood vessels and glial cells, which complicates neuronal segmentation by generating a complex background, and (2) imaging signal discontinuities due to the non‐uniform staining, which hinder the complete tracing of neuronal branches [24, 25]. To solve these problems, we designed a semi‐automated single‐neuron reconstruction method, SNR‐Golgi, which includes three key modules: background denoising, single‐neuron extraction, and branch repair. These modules enable efficient neuronal extraction and the repair of interrupted branch signals, significantly improving reconstruction completeness (Figure 1A). Using the fMOST dataset of Golgi‐stained neurons in the mouse VIS, we demonstrate the workflow of SNR‐Golgi (Figure 1B,C).

To address the complex background in Golgi‐stained samples, we first established a background denoising module. We observed distinct grayscale intensity differences between neurons and surrounding tissue, enabling the partial removal of background noise through an intensity threshold. We systematically evaluated the impact of various threshold values on the background removal by setting thresholds at 160, 190, 220, and 250, respectively (Figure 1D, Figure S1). Thresholds of 160 and 190 preserved most neuronal structures but left significant background noise and artefacts. Setting the threshold at 250 effectively removed the background but also lost critical neuronal structural details. The threshold of 220 balanced background noise reduction with preservation of neuronal structures, making it the optimal threshold for denoising. Next, we developed the single‐neuron extraction module. Despite background denoising, some high‐intensity punctate artefacts remained, which we addressed by applying a volume threshold (Figure 1E). Artefacts smaller than 1000 μm^3^ were removed, along with branches and blood vessels disconnected to the target neuron, to extract the target single neuron (Figure 1E). Finally, to address neuronal branch discontinuities, we established a branch repair module. We identified two major types of signal discontinuities: (1) mid‐branch interruptions and (2) undetected branches, primarily caused by staining inconsistencies (Figure 1F). Seed points were placed at the neuronal soma, and terminal points of interrupted branches were manually marked. Using the tracing algorithm, we repaired both types of signal discontinuities. 3D visualisation results confirmed effective repair of branch continuity, significantly enhancing reconstruction completeness (Figure 1F, Figure S2).

**FIGURE 2:**
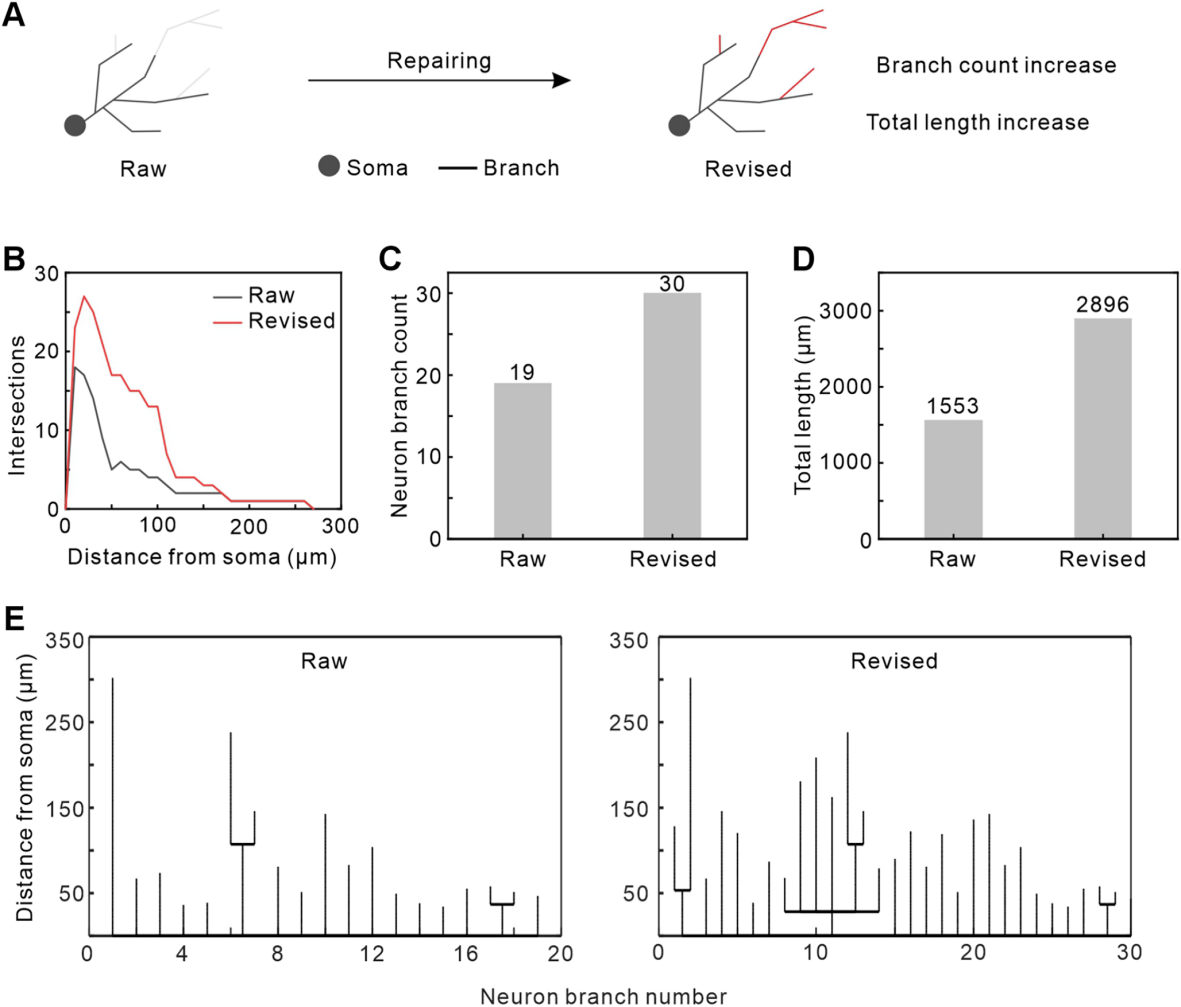
Analysis of neuronal branch repair using the SNR‐Golgi method. (A) Schematic illustration of neuronal branch repair. (B) Sholl analysis of the raw and revised neurons. (C) Branch count of the raw and revised neurons. (D) Total branch length of the raw and revised neurons. (E) Length distribution of individual neuronal branches of the raw and revised neurons.

To assess the effectiveness of neuronal branch signal repair, we quantified and analysed the morphological features of the raw and revised neurons, including neuronal complexity, branch number, and total branch length (Figure 2A). Neuronal complexity was evaluated using 3D Sholl analysis, in which a series of concentric spheres centred on the soma were used to count the number of intersections between the branches and spheres [26, 27]. After repair, the number of intersections significantly increased within 20–150 μm from the soma, indicating improved neuronal integrity and complexity (Figure 2B). Statistical analysis revealed that the total number of neuronal branches increased from 19 to 30, a 58% increase (Figure 2C,E). Additionally, the total branch length increased from 1553 μm to 2896 μm, an 86% improvement (Figure 2D,E). These findings demonstrate that SNR‐Golgi significantly enhances neuronal reconstruction integrity in Golgi‐stained samples, validating its reliability and effectiveness.

### 2.2 SNR‐Golgi for Single‐Neuron Reconstruction Across Brain Regions via fMOST

**FIGURE 3:**
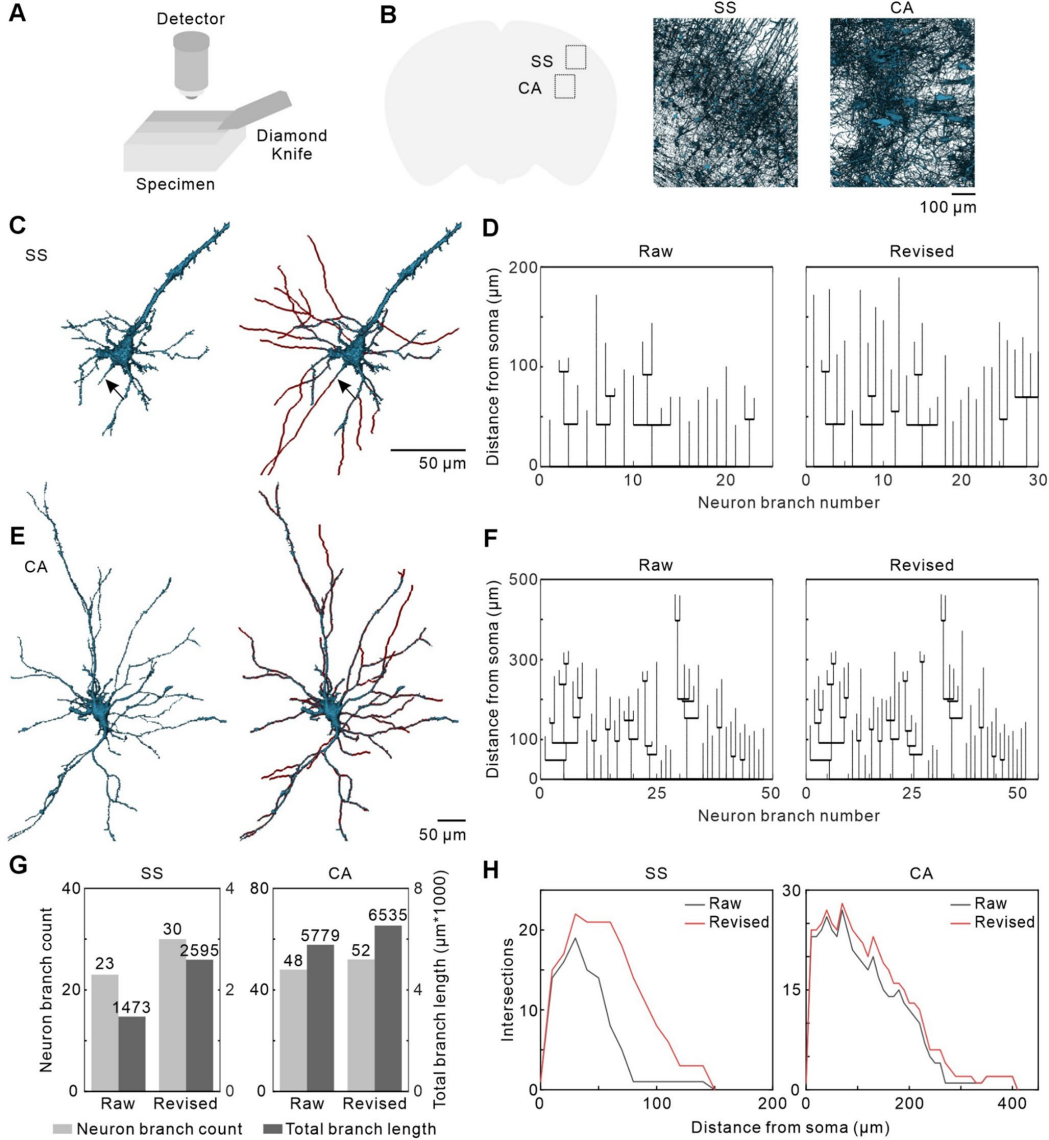
Analysis of single‐neuron extraction and repair across different brain regions using the SNR‐Golgi method. (A) Schematic representation of fMOST imaging. (B) Background‐denoised data from the SS and CA brain regions. (C) The raw and revised neurons in the SS region. The arrows indicate the axons. (D) Length distribution of individual neuronal branches in (C) of the raw and revised neurons. (E) The raw and revised neurons in the CA region. (F) Length distribution of individual neuronal branches in (E) of the raw and revised neurons. (G) Branch count and total branch length of the raw and revised neurons in the SS and CA regions. (H) Sholl analysis of the raw and revised neurons in the SS and CA regions.

To evaluate the applicability of SNR‐Golgi in single‐neuron extraction and reconstruction across different brain regions, we applied it to neurons in the SS and CA. fMOST imaging was performed at a voxel size of 1 μm × 1 μm × 2.5 μm, and a 300 μm‐thick coronal section was extracted for analysis (Figure 3A). First, the background denoising module effectively reduced tissue background, yielding a clear neuronal visualisation with low‐intensity background (Figure 3B). Using the single‐neuron extraction module, we successfully isolated individual neurons from both regions. 3D reconstructions revealed typical pyramidal neuron morphology, including the soma, basal dendrites, and apical dendrites (Figure 3C,E). However, in the SS region, basal dendrites appeared relatively short, and the axon (black arrows) remained incomplete, indicating incomplete reconstruction at this stage. Following application of the branch repair module, neuronal morphology was significantly improved in both regions, with a notable increase in axon length (arrows in Figure 3C,E), demonstrating the effectiveness of SNR‐Golgi in enhancing neuronal reconstruction (Figure 3C,E).

To quantify the impact of SNR‐Golgi on neuronal morphology, we analysed key structural features of the raw and revised neurons, including branch count, dendritic length, and neuronal complexity. In the SS and CA regions, total branch count increased from 23 to 30 (+30%) and from 48 to 52 (+8%), respectively (Figure 3D,F,G). Similarly, total dendritic length increased from 1473 μm to 2595 μm in the SS region (+76%) and from 5779 μm to 6535 μm in the CA region (+13%) (Figure 3G). Notably, axon length in the SS region exhibited a substantial increase from 47 μm to 172 μm, representing a 266% increase (Figure 3D). 3D Sholl analysis further revealed a significant increase in branch intersections within 30–150 μm from the soma in SS neurons, indicating improved neuronal integrity and complexity after repair (Figure 3H). These results demonstrate the broad applicability of SNR‐Golgi across different brain regions, highlighting its effectiveness in neuronal reconstruction.

### 2.3 SNR‐Golgi for Single‐Neuron Reconstruction via Synchrotron‐Based X‐Ray Micro‐CT

**FIGURE 4:**
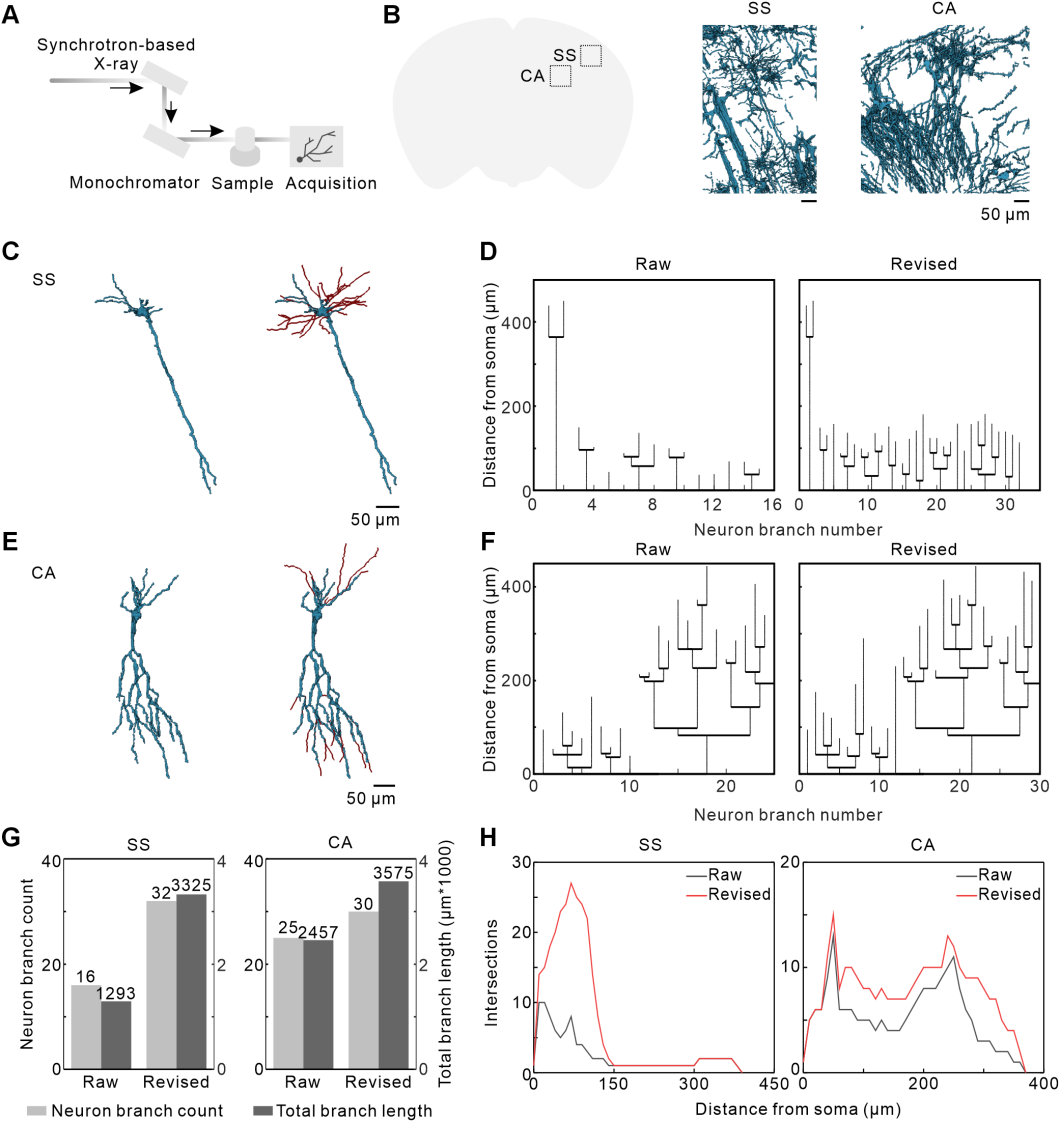
Analysis of single‐neuron extraction and repair across different imaging techniques using the SNR‐Golgi method. (A) Schematic representation of synchrotron‐based X‐ray Micro‐CT imaging. (B) Background‐denoised data from the SS and CA brain regions. (C) The raw and revised neurons in the SS region. (D) Length distribution of individual neuronal branches in (C) of the raw and revised neurons. (E) The raw and revised neurons in the CA region. (F) Length distribution of individual neuronal branches in (E) of the raw and revised neurons. (G) Branch count and total branch length of the raw and revised neurons in the SS and CA regions. (H) Sholl analysis of the raw and revised neurons in the SS and CA regions.

To further evaluate the applicability of SNR‐Golgi across datasets of different imaging techniques, we applied it to synchrotron‐based X‐ray micro‐computed tomography (Micro‐CT) datasets of Golgi‐stained mouse brain tissue. Different from conventional optical imaging techniques, X‐ray Micro‐CT offers high penetration depth, enabling the imaging of millimetre‐thick brain tissue without the need for thin physical sectioning, thereby preserving sample integrity [28, 29, 30, 31, 32]. Golgi‐stained mouse brains were coronally sectioned into 2‐mm‐thick slices, embedded in paraffin, and imaged using X‐ray Micro‐CT. Data acquisition was performed at a voxel size of 0.65 μm × 0.65 μm × 0.65 μm, with 1800 projections collected at equal angular intervals over a 0°–180° range for both the SS and CA regions. Following tomographic reconstruction, high‐resolution virtual slices of these brain regions were obtained (Figure 4A).

Subsequently, we applied the SNR‐Golgi for single‐neuron extraction and repair in the SS and CA regions. The background denoising module was first used to remove tissue background, producing clear visualisations (Figure 4B). For X‐ray Micro‐CT data, we evaluated the background denoising effects under different grayscale thresholds (20, 40, 60 and 80) and determined an optimal grayscale threshold of 40, which balanced background noise reduction with preservation of neuronal structures (Figure S3). Using the single‐neuron extraction module, we successfully isolated individual neurons from both regions. 3D reconstructions revealed characteristic pyramidal cell morphology in the SS and CA regions (Figure 4C,E). Following branch repair, neuronal structures in both regions exhibited markedly improved continuity and completeness (Figure 4C,E).

To further assess the impact of SNR‐Golgi on neuronal reconstruction, we quantitatively analysed morphological features before and after branch repair, including branch number, total branch length, and neuronal complexity. After repair, the total number of branches increased from 16 to 32 in the SS region and from 25 to 30 in the CA region, representing 100% and 20% increases, respectively (Figure 4D,F,G). Similarly, the total branch length increased from 1293 μm to 3325 μm in the SS region and from 2457 μm to 3575 μm in the CA region, corresponding to 157% and 46% increases, respectively (Figure 4G). 3D Sholl analysis revealed a significant increase in intersections within 20–150 μm from the soma in SS region neurons, whereas CA region neurons exhibited a marked rise in the length of longest observed branches, indicating enhanced neuronal complexity (Figure 4H). These findings confirm that SNR‐Golgi is broadly applicable across different imaging techniques and offers distinct advantages in reconstructing complex neuronal morphologies.

## 3 Conclusion

In summary, to address the challenges of neuronal segmentation and branch signal discontinuities in the 3D reconstruction of Golgi‐stained samples, we developed the SNR‐Golgi method. This approach integrates three key modules—background denoising, single‐neuron extraction, and branch restoration—significantly improving the completeness and accuracy of neuronal reconstruction. Validation using fMOST and synchrotron‐based X‐ray Micro‐CT datasets demonstrates the broad applicability of SNR‐Golgi across different brain regions and imaging modalities.

SNR‐Golgi offers several advantages. First, it enables efficient reconstruction in complex backgrounds. In Golgi‐stained samples with high labeling density and intricate background structures, SNR‐Golgi accurately extracts single neurons and restores interrupted branches, ensuring high‐fidelity neuronal reconstruction. Second, it exhibits strong adaptability across brain regions. The method successfully reconstructs neurons in diverse areas, including the VIS, SS, and hippocampal CA regions, supporting the study of region‐specific neuronal morphology and its role in functional networks. Third, it demonstrates cross‐modality robustness. SNR‐Golgi performs consistently across fMOST and synchrotron‐based X‐ray Micro‐CT datasets, indicating its capability to accommodate varying resolutions and imaging conditions. This adaptability provides a critical foundation for multimodal and cross‐platform neuronal imaging studies.

Despite these advantages, SNR‐Golgi has certain limitations that require further optimisation. Its artefact removal strategy currently relies on threshold‐based segmentation and volume filtering, which may be insufficient for high‐artefact datasets, particularly those with complex, high‐intensity noise. Integrating deep learning models for artefact recognition could enhance automation and improve removal accuracy. Additionally, the current reconstruction workflow requires manual intervention, limiting efficiency in large‐scale data processing. The development of fully automated approaches will be essential to enabling high‐throughput neuronal reconstruction and large‐scale morphological analyses.

## 4 Materials and Methods

### 4.1 Animals

Mice: C57BL/6 mice (6‐8‐week‐old, male) were purchased from Shanghai SLAC Laboratory Animal Co. Ltd., China. All the animal experiments were conducted in accordance with the Institute's Guide for the Care and Use of Laboratory Animals and were approved by the ethical committee of Shanghai Beautiful Life Medica Technology (approval no. SYXK‐2017‐0016, approved on 25 December 2017).

### 4.2 Golgi Staining

Mice underwent intracardiac perfusion with normal saline prior to brain extraction. Following removal from the skull, brains were promptly rinsed with normal saline to eliminate residual blood from the surface. Neuronal Golgi impregnation was carried out using the Hito Golgi‐Cox OptimStain Kit. For the Golgi‐Cox method, the samples were stained under dark conditions at 26°C.

### 4.3 fMOST Imaging

After Golgi‐Cox staining of the mouse brain, residual Golgi solution was removed by rinsing the tissue with deionised water. The brain tissue was then subjected to development with a 1% lithium hydroxide solution. Following gradient ethanol dehydration, the brain tissue was embedded using resin embedding techniques. Imaging was subsequently performed with the fMOST system (Biomapping 5000) at a voxel size of 1 μm × 1 μm × 2.5 μm.

### 4.4 Synchrotron‐Based X‐Ray Micro‐CT Imaging

After Golgi‐Cox staining, the mouse brain tissue was preserved using a 30% sucrose solution. Developing was performed with a mixture of Solution 4 and Solution 5 from the Hito Golgi‐Cox OptimStain Kit. Following gradient ethanol dehydration and xylene clearing, the tissue was embedded using paraffin embedding techniques. Synchrotron‐based X‐ray Micro‐CT imaging was conducted at the 16 U2 beamline of the Shanghai Synchrotron Radiation Facility. The X‐ray energy was set to 19.3 keV, with an exposure time of 200 ms per projection. The voxel size achieved was 0.65 μm × 0.65 μm × 0.65 μm, and 1800 projections were acquired over a 0°–180° rotation at equal angular intervals.

### 4.5 Micro‐CT Reconstruction and Visualisation

The projection data were reconstructed into slice dataset using MOCUPY software. After determining the rotation centre of the sample, the Gridrec algorithm was applied to convert the projection data into virtual slices. The virtual slices were then preprocessed in Fiji ImageJ, including cropping to remove circular boundaries and converting the data to 8‐bit slices. Finally, 3D visualisation was performed using the Volume Rendering module in Amira software.

### 4.6 Single‐Neuron Extraction and Repair

In Amira software, the region containing the target single neuron was cropped. Threshold segmentation was performed on the selected region using the Interactive Thresholding module. The Remove Small Spots module was applied to eliminate small artefact points, while the Analysis Filter module was used for filtering to automatically remove partial interfering neuronal branch structures. Residual interfering branches were manually removed using the Volume Edit module, enabling 3D extraction of the target single neuron. Seed points and markers were then placed at the soma and branch termini of the neuron, respectively. The Filament module was used to repair fragmented neuronal branches, and results were exported in SWC format. The SWC files were processed using MATLAB and Python scripts to calculate parameters such as the number of neuronal branches and total branch length of the raw and revised neurons. Additionally, MATLAB was used to analyse the intersection of the neuron with concentric spheres based on the SWC data.

## Author Contributions

CRediT: Ying Zhu supervised the research. Qiaowei Tang, Binfu Fan, and Xiaoqing Cai performed experiments. ZhiMing Shen helped in the guidance of data analysis. Jichao Zhang helped in the guidance of X‐ray microscopy. Jun Hu and Jiang Li performed critical revisions. Qiaowei Tang, Binfu Fan, and Ying Zhu analysed the data and wrote versions of the manuscript. All authors discussed and commented on the manuscript.

## Conflicts of Interest

The authors declare no conflicts of interest.

## Supporting information


**Data S1.** Supporting Information.

## Data Availability

The data that support the findings of this study are available from the corresponding author upon reasonable request.
