# Supplementary material for: High‐Resolution Single‐Neuron Reconstruction Analysis in Golgi‐Stained Brain Tissues
Source: Cell Prolif. 2025 Jul 6;59(2):e70092. doi: 10.1111/cpr.70092 (PMC12877961; doi:10.1111/cpr.70092)
Supplement: Supplementary file 1 — Data S1. Supporting Information. [file CPR-59-e70092-s001.docx]

**Supporting information**

**High-resolution single-neuron reconstruction analysis in Golgi-stained brain tissues**

Qiaowei Tang^1,2#^, Binfu Fan^3#^, Xiaoqing Cai^4#^, Zhiming Shen^5^, Jichao Zhang^4^, Jun Hu^1,3^, Jiang Li^1^, Ying Zhu^1*^

^1^Institute of Materiobiology, College of Sciences, Shanghai University, Shanghai 200444, China

^2^Xiangfu Laboratory, Jiashan 314102, China

^3^School of Physical Science and Technology, ShanghaiTech University, Shanghai 201210, China

^4^Shanghai Synchrotron Radiation Facility, Shanghai Advanced Research Institute, Chinese Academy of Sciences, Shanghai 201210, China

^5^Institute of Neuroscience, Key Laboratory of Brain Cognition and Brain-inspired Intelligence Technology, Center for Excellence in Brain Science and Intelligence Technology, Chinese Academy of Sciences, Shanghai 200031, China

*E-mail: zhuying331@shu.edu.cn

**Supporting Information:**

**Supporting Table S1.** Branch count and total branch length of neurons before and after repair

**Supporting Figures S1-S3.**

**Table S1.** Branch count and total branch length of the raw and revised neurons.

| Techniques | Regions | The number of branches | | The total length of branches (μm) | |
| --- | --- | --- | --- | --- | --- |
|  |  | Before | After | Before | After |
| fMOST | VIS | 19 | 30 | 1553 | 2896 |
|  | SS | 23 | 30 | 1473 | 2595 |
|  | CA | 48 | 52 | 5779 | 6535 |
| SR X-ray Micro-CT | SS | 16 | 32 | 1293 | 3325 |
|  | CA | 25 | 30 | 2457 | 3575 |


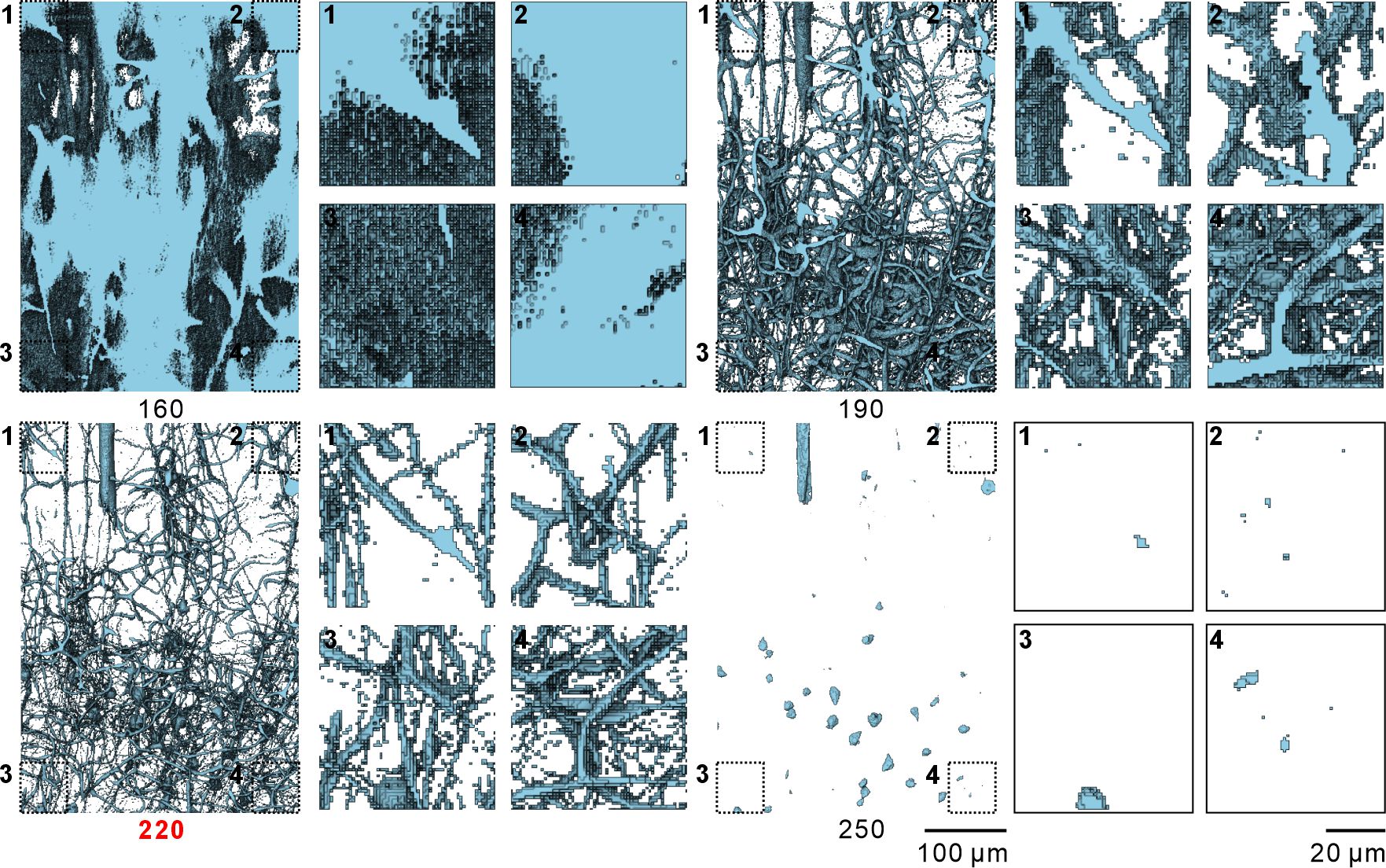


**Figure S1.** Visualization and magnified views of the VIS region at different grayscale thresholds of fMOST dataset.


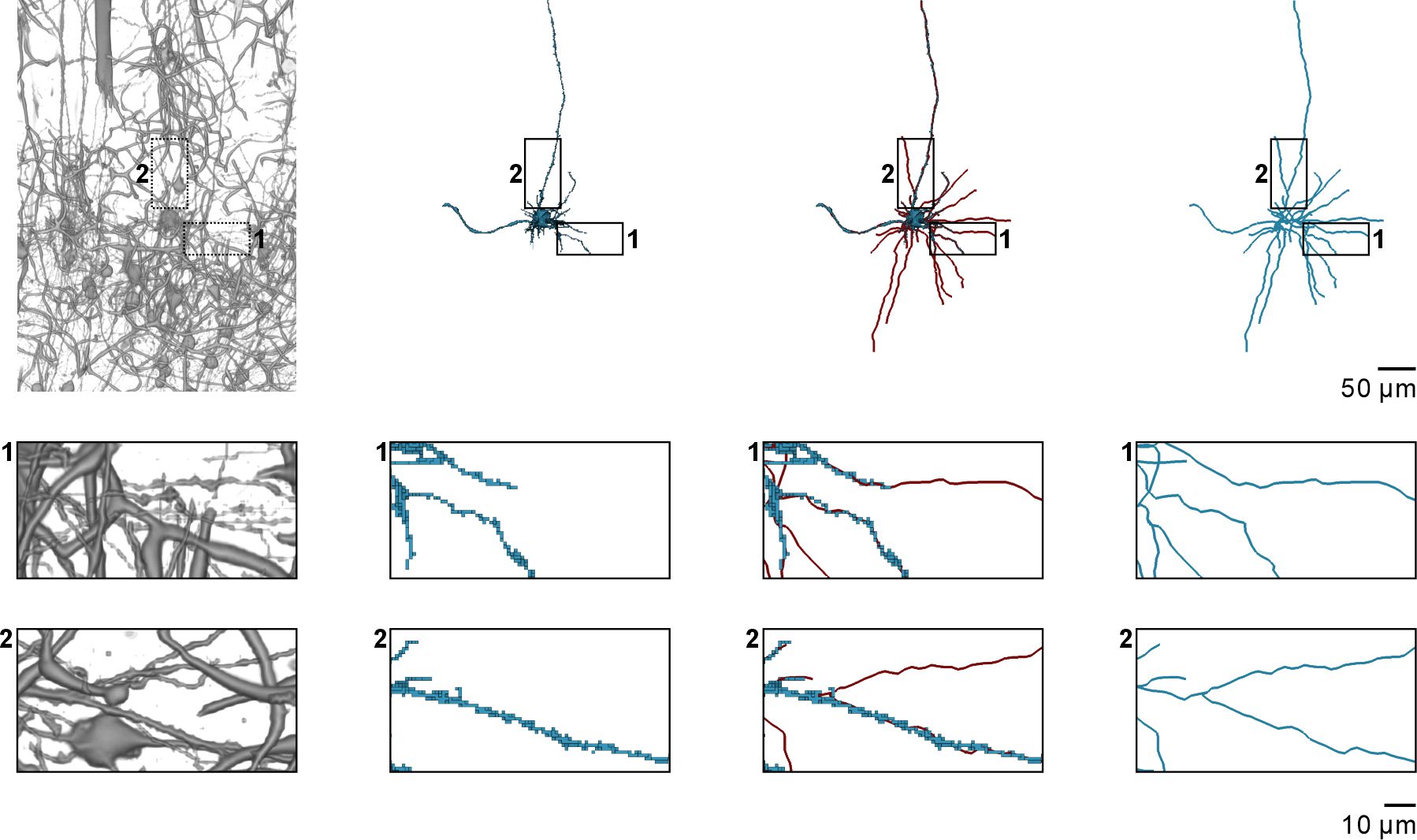


**Figure S2.** Branch repair module for repairing two types of branch signal discontinuities. From left to right: raw image, extracted single-neuron image, single-neuron image during repairing, and repaired single-neuron image.


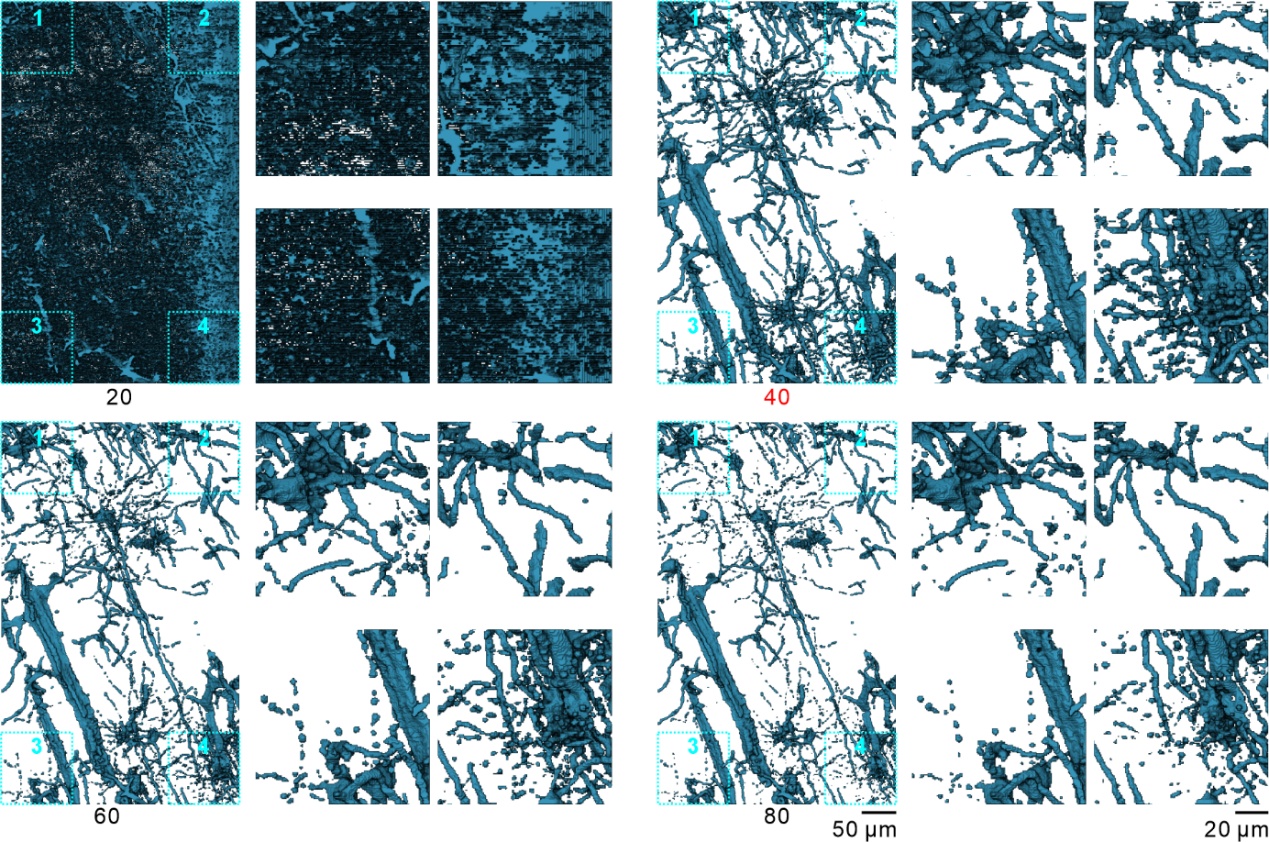


**Figure S3.** Visualization and magnified views of the SS region at different grayscale thresholds of Micro-CT dataset.
